# A Rare Case of Budd–Chiari Syndrome in a Young Female

**DOI:** 10.1002/ccr3.70478

**Published:** 2025-05-04

**Authors:** Joshua Chacko, Raymond Haward, Newton Ashish Shah, Saravenensandeep V. Pathmanathan, Bharath Sundaramoorthy, Jyothi Halepalya Tukaram

**Affiliations:** ^1^ Father Muller Medical College General Medicine Bangalore India; ^2^ Vydehi Institute of Medical Sciences and Research Centre Bangalore India; ^3^ Tribhuvan University Teaching Hospital Kathmandu Nepal; ^4^ Medical University of Lodz Lodz Poland; ^5^ Government Thoothukudi Medical College Hospital Thoothukudi India; ^6^ Norman Bethune Medical University Changchun China

**Keywords:** Budd–Chiari syndrome, gastrology, hepatic veins, portal hypertension, thrombosis

## Abstract

Budd–Chiari syndrome is a rare disorder characterized by hepatic vein obstruction, with an incidence of 1 in 100,000. It can be diagnosed through imaging studies and initially managed with anticoagulants. Acute Budd–Chiari syndrome can be treated with thrombolysis to dissolve blood clots obstructing hepatic veins. Venous obstructions in Budd–Chiari syndrome can be fixed through angioplasty or venous stenting to restore blood flow. Liver decompression can be achieved with TIPS, and severe cases may require liver transplantation.

## 1 Introduction

Budd–Chiari syndrome (BCS) is a rare disorder caused by the constriction of hepatic venous outflow, involving at least two hepatic veins [1]. This obstruction occurs without cardiac involvement. Dr. George Budd in 1846 reported three incidences of hepatic vein blockage. Approximately five decades later, Dr. Hans Chiari provided a detailed pathological and clinical overview of the disease [2]. Budd–Chiari syndrome can be classified as primary or secondary depending on the involvement of the hepatic veins, primarily the inferior vena cava (IVC), or external factors like neoplasm, respectively [3]. The incidence and prevalence of BCS are very scarce. According to a systemic review and meta‐analysis, the incidence of BCS is 1 in 100,000, and the prevalence is 11 in 100,000 [4]. Females in Western nations exhibit a higher prevalence of BCS, while the Asian population displays contrasting patterns [5]. Budd–Chiari syndrome can impact individuals of any age; however, it is frequently observed in individuals between 30 and 50 years [6].

Myeloproliferative diseases, factor V Leiden, and protein C deficiency contribute to approximately 50%, 25%, and 12.5% of the overall BCS cases, respectively [5]. Other clinically relevant etiologies of BCS include malignancy (10% cases), liver lesions, pregnancy, and the use of oral contraceptive pills (20% cases) [3]. Mutations in the Janus tyrosine kinase‐2 (JAK2) gene account for 37%–57% of primary BCS cases [7]. Hepatic venous occlusion causes interstitial fluid infiltration, resulting in liver congestion, impaired lymphatic drainage, and fluid accumulation in the abdomen (ascites). This blockade of blood flow in the veins causes hepatic portal venous hypertension. If left untreated, this can exaggerate, leading to fibrosis, cirrhosis, and hepatocellular cancer, and it can even be fatal [3]. The clinical presentation of BCS can vary from being asymptomatic (15%–20%) to subacute or chronic (60%), acute (20%), and fulminant (5%) [8]. Classical BCS manifests as sudden and severe abdominal pain, ascites, and hepatomegaly [9, 10]. Budd–Chiari syndrome may also present as persistent abdominal discomfort and varicose veins in the abdominal wall [9]. Patients also report altered liver function test (LFT) values [10]. Therefore, it is important to recognize and treat BCS promptly. Although there have been improvements in understanding BCS, there are still deficiencies in identifying its symptoms and adopting timely and effective treatment approaches.

This case demonstrates the clinical course, diagnosis, and therapeutic response of a 20‐year‐old student with acute BCS, managed with the anticoagulant heparin. The present case underscores the importance of a thorough and appropriate diagnosis.

## 2 Case History/Examination

A 20‐year‐old female patient with no previous comorbidities presented with abdominal pain for 1 week. The pain was colicky and localized to the right upper quadrant, without radiation. Over the week, the pain was accompanied by progressive abdominal distention. She experienced non‐bilious, non‐bloody vomiting 1–2 times per day. Additionally, she had a low‐grade fever for the past 2 days, without chills or rigors. She reported no history of loose stools, melena, jaundice, sleep disturbances, or tuberculosis.

On general examination, her pulse rate was 69 beats per minute, her blood pressure was 110/70 mmHg (supine), and her temperature was 99.2°F. She exhibited pallor and pitting edema but no icterus, koilonychia, or lymphadenopathy. There were no signs of flapping hand tremors, parotid enlargement, or spider nevi.

Per abdomen examination on inspection revealed a distended abdomen with full flanks and a stretched, centrally located, and inverted umbilicus. There were no dilated veins, scars, or hernias, and the orifices appeared normal. Palpation confirmed the inspection findings and revealed generalized tenderness, especially in the right upper quadrant, with no palpable liver or spleen. Percussion revealed the presence of shifting dullness and fluid thrills, indicating the presence of ascites. Auscultation detected 3–5 bowel sounds per minute, and no bruits were heard. Respiratory examination revealed dullness on percussion in the bilateral infraaxillary and infrascapular areas. Decreased vocal resonance and vocal fremitus were also noted in these regions. The cardiovascular system examination was normal, except for an ejection systolic murmur heard in the aortic area.

## 3 Methods

Table 1 shows the patient's blood workup on hemogram analysis, coagulation profile, liver function test, and amylase test. Our patient was notable for elevated alanine (746) and aspartate transaminase (1237), conjugated bilirubin and elevated INR (2.16) and prothrombin ratio (24.2). The patient had low hemoglobin (6.8), platelets (115,500) and serum albumin (3.03) correlating with the diagnosis. Tables 2 and 3 show physical and biochemical evaluations of the ascitic fluid. MRI findings suggested attenuation of the IVC drainage from the entire course of the right, middle, and left hepatic veins and dilated portal vein with portosystemic collateral as seen in Video 1. The differential diagnosis of the patient was either non‐cirrhotic portal hypertension, acute viral hepatitis, and extrahepatic portal vein obstruction (EHPVO). Given her clinical presentation, medical history, and diagnostic evaluations, her primary diagnosis was Budd–Chiari syndrome.

**TABLE 1:** Laboratory investigation results.

Investigations	Patient findings	Normal reference ranges
Hemoglobin (g/dL)	6.8^a^	12.0–15.5
Leukocyte (cells/μL)	**12,500**	4000‐11,000
Neutrophils (%)	55.3%	40%–60%
Eosinophils (%)	1%	1%–4%
Basophils (%)	0.7%	0.5%–1%
Lymphocyte (%)	**42%**	20%–40%
Monocyte (%)	1%	2%–8%
Erythrocyte sedimentation rate (mm/h)	**22**	0–20
Platelets (cells/μL)	115,500^a^	150,000‐450,000
Mean corpuscular volume (fL)	62^a^	80–100
Mean corpuscular hemoglobin (pg)	17.9^a^	27–32
Mean corpuscular hemoglobin concentration (g/dL)	28.9^a^	32–36
Prothrombin time (s)	**24.2**	11–13.5
International normalized ratio	**2.16**	0.8–1.2
Partial thromboplastin time (s)	29.9	25–35
Serum total protein (g/dL)	5.21	6.0–8.3
Serum albumin (g/dL)	3.03^a^	3.5–5.0
Serum globulin (g/dL)	2.2^a^	2.3–3.5
Serum A/G ratio	1.4	1.1–2.5
Serum total bilirubin (mg/dL)	**1.26**	0.1–1.2
Serum conjugated bilirubin (mg/dL)	**0.75**	0.0–0.3
Serum unconjugated‐bilirubin (mg/dL)	0.51	0.1–1.0
Serum aspartate aminotransferase (U/L)	**746**	10–40
Serum alanine aminotransferase (U/L)	**1237**	7–56
Serum alkaline phosphatase (U/L)	61	44–147
Amylase (U/L)	19.9	23–85
Lipase (U/L)	16	0–160
HbsAg	Negative	Negative
Hepatitis C antibody (anti‐HCV) test	Negative	Negative
Blood urea nitrogen (mg/dL)	9.24	7–20
Creatinine (mg/dL)	0.7	0.6–1.1

^a^ Indicates lower values, bold text indicates higher values.

**TABLE 2:** Ascitic fluid analysis.

Ascitic fluid analysis	Patient findings	Significance
Color	Pale yellow	Normal finding
Appearance	Clear pale yellow	Normal finding
Coagulum	Not seen	Normal finding
Total count by microscopy	550 cells/cumm	Elevated (normal < 500 cells/cumm)
Differential cell count neutrophils	10%	Normal
Differential cell count lymphocytes	90%	Normal

**TABLE 3:** Ascitic fluid biochemical report.

Ascitic fluid biochemical	Patient findings	Normal reference ranges
Albumin (g/dL)	0.91 (SAAG‐2.12)	SAAG < 1.1 g/dL
Ascitic fluid glucose (mg/dL)	131	50–100
Ascitic fluid protein (g/dL)	1.57	< 2.5 (transudative) or > 2.5 (exudative)
Ascitic fluid chloride (mmol/L)	111.5	102–109
Ascitic fluid amylase (U/L)	38.8	< 85
Ascitic fluid LDH (U/L)	3784	< 225
Ascitic fluid adenosine deaminase (U/L)	2.6	< 40

**VIDEO 1:** MRI shows attenuation of the IVC drainage, dilated portal vein with portosystemic collateral, along with hypertrophy of caudate lobe. Video content can be viewed at https://onlinelibrary.wiley.com/doi/10.1002/ccr3.70478

The patient was treated symptomatically. The patient's peripheral smear came back with severe microcytic hypochromic anemia, and the SAAG came out to be 2.12, which is significant for portal hypertension. We started her on standard anticoagulant therapy of heparin (1 mg/kg/day) for 1 week, along with diuretics. Serial endoscopy findings were unremarkable. Screening for causes of thrombophilia (protein C, Protein S, Antithrombin, Factor V, Lupus anticoagulant, and Antiphospholipid antibodies) came back negative.

## 4 Conclusion and Results

The patient showed improvement after 12 days of admission, leading to their discharge from the hospital. We transitioned the patient to warfarin and instructed him to continue for 3 months. A 2‐week follow‐up revealed that the patient was asymptomatic and the blood work came back normal. We also started the patient on iron supplementation with ferrous sulphate (325 mg) twice a day for 3 months. We advised the patient on the importance of taking supplements on an empty stomach 1–2 h before a meal. During the follow‐up in the third month, the patient had a normal liver function test, and the complete blood count still showed findings of iron deficiency anemia. On doing a serial upper GI endoscopy was unremarkable for varices. Warfarin was recommended to continue indefinitely with strict monitoring of PT/INR at least once every 6 months.

## 5 Discussion

This case of a 20‐year‐old female student presenting with BCS highlights the relevance of prompt diagnosis and appropriate treatment of this life‐threatening, rare disease. Budd–Chiari syndrome manifests from the obstruction of venous outflow of the liver at any site from the hepatic venules to the IVC into the right atrium. It is the obstruction of two or more significant hepatic veins that leads to elevated pressure in the sinusoids and decreased blood flow through them. This condition manifests as BCS clinical presentation [11]. The patient's symptoms of stomach discomfort, distension, vomiting, and fever led to a clinical suspicion of BCS.

Clinical presentation and confirmatory investigations help in the diagnosis of BCS. In the present case, the presence of abdominal pain, abdominal distension with fluid thrill, shifting dullness, and tenderness in the abdomen toward the right corroborated the hallmark features of BCS: abdominal pain and distension, ascites, and hepatomegaly [12]. Respiratory findings like decreased vocal resonance and breath sounds pointed to ascites and possibly pleural effusions due to blocked hepatic veins. However, studies have suggested that approximately 15% of BCS cases have no conventional symptoms and are usually identified on suspicion of chronic liver disease or unusual LFT values [13, 14]. Abnormal LFT is a prominent characteristic; however, it may be within the normal range for certain people. In cases of fulminant and acute BCS, elevations in serum ALT and AST may exceed five times the upper limit of normal. If any of these results are present, it is necessary to do additional analyses to rule out BCS. For confirmatory diagnosis, radiological imaging should be performed even in cases with minimal clinical suspicion [8, 15, 16]. In our case, radiological evaluations confirmed the presence of clear, pale yellow ascitic fluid without clots, supporting the diagnosis of BCS.

Invasive hepatic venography and cavography are widely regarded as the most reliable methods for diagnosing BCS. Nevertheless, these invasive angiographic methods are not appropriate for the routine diagnosis of suspected clinical situations due to their unavailability, high cost, and potential consequences [17]. According to the Asian Pacific Association for the Study of the Liver (APASL) recommendations, imaging techniques like Doppler ultrasound are the first line of BCS diagnosis [17]. Doppler ultrasound can visualize blood flow and detect blood clots within the liver [18]. To confirm BCS, estimate the degree of thrombosis, rule out cancer, evaluate hepatic nodules, and analyze hepatic morphological modifications, contrast‐enhanced computed tomography (CECT) or magnetic resonance imaging (MRI) can be performed [17]. Patients with BCS have an increased susceptibility to develop hepatocellular carcinoma. Surveillance utilizing ultrasonography and serum alpha‐fetoprotein levels is advised, although it is not the most effective approach [17]. Assessing portal hypertension, analyzing the ascitic fluid, performing upper gastrointestinal endoscopy to evaluate varices, and investigating thrombosis are all critical components of the diagnostic process for suspected BCS patients [19].

Budd–Chiari syndrome can be managed either using anticoagulation therapy or endovascular therapy and surgery. Guidelines recommend anticoagulation therapy as a first‐line treatment. Low molecular weight heparin should be given to patients for 2 weeks to 2 months along with symptomatic treatment [17]. However, the risk of anticoagulation should be considered, especially in patients with bleeding complications or varices. Before initiating anticoagulation, an upper endoscopy can be performed to screen for varices. Endovascular intervention can be used for BCS to alleviate the blockage of hepatic venous outflow [19]. Transjugular Intrahepatic Portosystemic Shunt (TIPS) is the preferred treatment for BCS in cases where there is portal hypertension that does not improve with conventional therapy [17]. Pharmacological thrombolysis, surgery, and liver transplantation can be considered in acute cases of BCS [17, 19].

## 6 Conclusion

The present case highlights the need for appropriate and timely diagnosis in BCS patients. Individuals presenting with no conventional symptoms, chronic liver disease, or unusual LFT values must be evaluated further for BCS. Gaps in the literature exist on the early detection and differentiation of BCS from other causes of portal hypertension and hepatic dysfunction. Hence, future research should emphasize developments in diagnostic accuracy, advanced early diagnostic tools, and optimal therapeutic regimens to improve patient outcomes in BCS.

## Author Contributions

**Joshua Chacko:** conceptualization, data curation, formal analysis, funding acquisition, investigation, methodology, project administration, resources, software, supervision, validation, visualization, writing – original draft, writing – review and editing. **Raymond Haward:** conceptualization, data curation, formal analysis, funding acquisition, investigation, methodology, project administration, resources, software, supervision, validation, visualization, writing – original draft, writing – review and editing. **Newton Ashish Shah:** conceptualization, data curation, formal analysis, funding acquisition, investigation, project administration, resources, software, supervision, validation, visualization, writing – original draft, writing – review and editing. **Saravenensandeep V. Pathmanathan:** data curation, formal analysis, funding acquisition, methodology, project administration, software, supervision, validation, visualization, writing – original draft, writing – review and editing. **Bharath Sundaramoorthy:** conceptualization, data curation, formal analysis, funding acquisition, methodology, project administration, supervision, validation, visualization, writing – original draft, writing – review and editing. **Jyothi Halepalya Tukaram:** conceptualization, data curation, formal analysis, investigation, methodology, project administration, resources, supervision, validation, visualization, writing – original draft, writing – review and editing.

## Consent

Written informed consent was obtained from the patient for publication of this case report and any accompanying images. A copy of the written consent is available for review by the Editor‐in‐Chief of this journal on request.

## Conflicts of Interest

The authors declare no conflicts of interest.

## Data Availability

All the required information is within the manuscript itself.
